# Multiscale limited penetrable horizontal visibility graph for analyzing nonlinear time series

**DOI:** 10.1038/srep35622

**Published:** 2016-10-19

**Authors:** Zhong-Ke Gao, Qing Cai, Yu-Xuan Yang, Wei-Dong Dang, Shan-Shan Zhang

**Affiliations:** 1 School of Electrical Engineering and Automation, Tianjin University, Tianjin 300072, China

## Abstract

Visibility graph has established itself as a powerful tool for analyzing time series. We in this paper develop a novel multiscale limited penetrable horizontal visibility graph (MLPHVG). We use nonlinear time series from two typical complex systems, i.e., EEG signals and two-phase flow signals, to demonstrate the effectiveness of our method. Combining MLPHVG and support vector machine, we detect epileptic seizures from the EEG signals recorded from healthy subjects and epilepsy patients and the classification accuracy is 100%. In addition, we derive MLPHVGs from oil-water two-phase flow signals and find that the average clustering coefficient at different scales allows faithfully identifying and characterizing three typical oil-water flow patterns. These findings render our MLPHVG method particularly useful for analyzing nonlinear time series from the perspective of multiscale network analysis.

Uncovering complicated behavior from nonlinear time series constitutes a fundamental problem of continuing interest and it has attracted a great deal of attention from a wide variety of fields on account of its significant importance. Different methodologies have been developed to fulfill this challenging task, e.g., chaotic analysis^1^, fractal analysis^2^,^3^, recurrence plot^4^, complexity measure^5^, multiscale entropy^6^, and time-frequency representation^7^. In recent years, a new multidisciplinary methodology using complex network has emerged for characterizing complex systems^8^,^9^,^10^,^11^,^12^,^13^,^14^, especially the complex network analysis of time series, which has undergone a dramatic advance. Many efficient methods have been proposed to infer complex networks from univariate or multivariate time series^15^,^16^,^17^,^18^,^19^,^20^,^21^,^22^,^23^,^24^,^25^. In particular, Lacasa *et al.* proposed visibility graph^17^ and horizontal visibility graph^18^ which allow mapping a univariate time series into a complex network. The visibility graph theory has established itself as an efficient tool for probing the dynamics underlying real complex systems from time series^26^,^27^,^28^,^29^,^30^,^31^,^32^,^33^,^34^,^35^,^36^,^37^,^38^,^39^,^40^. The (horizontal) visibility graph leads to a natural graph-theoretical description of nonlinear systems with qualities in the spirit of symbolic dynamics. More recently, we extended the visibility graph to develop a limited penetrable visibility graph (LPVG)^41^,^42^ and found that LPVG presents a good anti-noise ability especially for the analysis of signals polluted by noise. Our LPVG method has been successfully applied to analyze gas-liquid flow signals^42^, signals from electromechanical system in process industry^43^, and EEG signals associated with manual acupuncture^44^ and Alzheimer’s disease^45^.

As a further study, we in this paper develop a novel multiscale limited penetrable horizontal visibility graph (MLPHVG) to analyze nonlinear time series from the perspective of multiscale and complex network analysis. In particular, we use two examples to demonstrate the validity of our method, i.e., (a) EEG signals recorded from healthy subjects and epilepsy patients; (b) experimental flow signals from oil-water two-phase flows.

The brain is one of the most complex systems. Epilepsy is a paroxysmal disorder of the brain, characterized by sudden occurrence of unprovoked seizures. The underlying mechanism of epileptic seizure is still elusive. Since the fluctuations of EEG signals are associated with the occurrence of epileptic seizures, the characterization of epileptic seizure from EEG signals becomes quite important. We apply our method to analyze two sets of EEG data recorded from numbers of healthy and epileptic subjects. We combine the support vector machine and network statistical measures including the average clustering coefficient and clustering coefficient entropy to detect epileptic seizures at different scales. We interestingly find that the network statistical measures present significant difference between healthy subjects and epilepsy patients. The classification accuracy is 100% at the scale factor 2. These results indicate that our method allows efficiently classifying and identifying EEG signals recorded from healthy subjects and epilepsy patients during epileptic seizures.

Liquid-liquid two-phase flows are widely encountered in many industrial processes. The mixture flow of immiscible oil-water can be viewed as a complex system with typical features of instability, transient and randomness. In recent years, the interest in oil-water two-phase flows has greatly increased due to the development of petroleum industry. The oil and water usually coexist during the oil-well production, and these two immiscible fluids can distribute themselves in various temporal-spatial configurations, known as flow patterns. Different flow patterns exhibit distinct local flow behaviors, how to identify and uncover the underlying dynamics of different flow patterns from experimental measurements has represented a challenge of significant importance. We carry out oil-water two-phase flow experiment to obtain the flow signals and then use our proposed method to identify and characterize different flow patterns from the experimental measurements. The results suggest that our method enables to identify distinct flow behaviors underlying three typical oil-water flow patterns. The above findings render our MLPHVG method particularly powerful for characterizing a dynamical process underlying a given nonlinear time series of time dependent complex system.

## Results

### MLPHVG analysis of EEG signals

**Figure 1:**
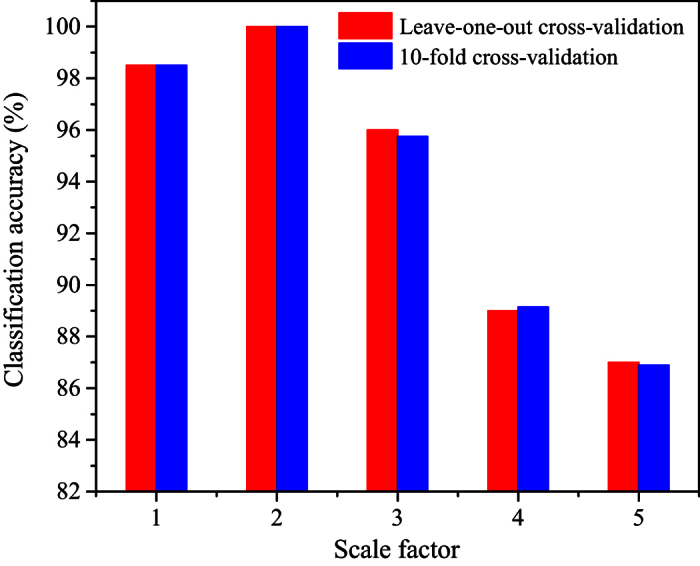


**Figure 2:**
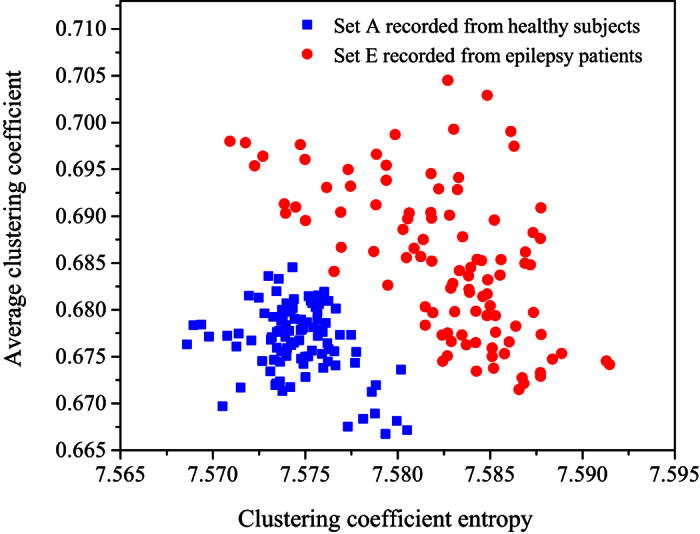


The EEG data sets analyzed in this paper are from the experiments carried out by Andrzejak *et al.*^46^. We use two EEG data sets (set A and set E) and each data set consists of 100 single-channel EEG segments of 23.6 s duration. These segments were selected and cut out from continuous multichannel EEG recordings after visual inspection for artifacts, e.g., due to muscle activity or eye movements. Set A consists of segments taken from five healthy subjects through surface electrodes using the international 10–20 electrode placement scheme. Set E from five epilepsy patients consists of segments selected from all recording sites exhibiting ictal activity during seizure activity. All EEG signals were recorded with the same 128-channel amplifier system, using an average common reference. The data were digitized at sampling rate of 173.61 Hz. Band-pass filter settings were 0.53–40 Hz. We derive 200 MLPHVGs (multiscale limited penetrable horizontal visibility graphs) corresponding to two sets of EEG signals with the limited penetrable distance being 1. Then we calculate the average clustering coefficient^47^ and clustering coefficient entropy^24^ from the derived networks. We combine the average clustering coefficient and clustering coefficient entropy to generate two-dimensional feature vectors and then employ SVM (Support Vector Machine) to realize the classification of sets A and E. In particular, we employ the leave-one-out cross-validation and 10-fold cross-validation to estimate the classified results of the features derived from MLPHVGs. The leave-one-out cross-validation^48^ consists of removing one sample from the dataset (set A and E), constructing the decision function on the basis only of the remaining dataset and then testing on the removed sample. In this fashion this process is repeated 200 times independently, with a different sample left out for testing every time. After 200 cross validations, we obtain the predicting labels for all samples and measure the fraction of correctly predicted samples over the total number of samples in the dataset. In addition, we employ the 10-fold cross-validation to estimate the classification accuracy. For one realization of 10-fold cross-validation, the 200 samples from sets A and E are randomly partitioned into ten equal subsets; nine subsets are used for training and one subset remains for testing. This procedure is repeated ten times so each subset serves once for validation and then we obtain predicting labels for all samples from ten subsets and a classification accuracy for one implementation of 10-fold cross-validation can be obtained by measuring the fraction of correctly predicted samples over the total number of samples in the dataset. In order to reduce bias introduced by randomly partitioning dataset in the cross-validation, we implement the 10-fold cross-validation 10 times independently and the final classification accuracy of set A and E can be estimated by taking the average of the 10 independent realizations of 10-fold cross-validation. The classification accuracy using leave-one-out cross-validation and 10-fold cross-validation at different scales are presented in Fig. 1. Notably, the classification accuracy is high over different scales and the highest value is 100% at scale 2. We in Fig. 2 show the joint distributions of the average clustering coefficient and clustering coefficient entropy for sets A and E at scale factor 2. This EEG database has been recognized as a benchmark for developing seizure detection models, and many researchers have used this database to test their proposed methods^49^,^50^,^51^,^52^,^53^,^54^,^55^,^56^. To give a few examples, Nigam *et al.*^49^ proposed a method using multistage nonlinear pre-processing filter in combination with a diagnostic artificial neural network to classify sets A and E with a classification accuracy of 97.2%. Kaya *et al.*^50^ presented a novel method based on one-dimensional local binary pattern to classify sets A and E and the classification accuracy is 99.5%. Kannathal *et al.*^51^ proposed a method based on various entropy measures and adaptive neuro-fuzzy classifier to classify sets A and E with a classification accuracy of 92.22%. Subasi^52^ employed wavelet feature extraction and a mixture of expert model to distinguish sets A and E and obtained a classification accuracy of 94.5%. Polat *et al.*^53^ classified sets A and E with a classification accuracy of 98.72% by using a hybrid system based on decision tree classifier and fast Fourier transform. Nicolaou *et al.*^54^ integrated the permutation entropy with the support vector machine to classify sets A and E with a classification accuracy of 93.55%. Zhu *et al.*^55^ proposed a weighted horizontal visibility graph to classify sets A and E and the classification accuracy is 100%. Zamir^56^ developed linear least squares-based preprocessing models to classify sets A and E with a classification accuracy of 100%. There are many published good classification results of this EEG dataset and the above are just a few of them. More results can be found in Ref. 57. Therefore, our method allows accurately classifying EEG signals recorded from healthy subjects and epilepsy patients.

### MLPHVG analysis of experimental flow signals

The two-phase flow signals are from our oil-water two-phase flow experiment, which was carried out in a vertical upward 20 mm-inner-diameter plexiglass pipe at Tianjin University. The experiential media are tap-water and No. 3 white oil. These two immiscible fluids mix themselves and then flow together into the vertical testing pipe. Three oil-in-water flow patterns have been observed, i.e., oil-in-water slug flow, oil-in-water bubble flow, oil-in-water VFD flow (Very Fine Dispersed bubble flow). The conductance sensor^58^ is designed to capture the flow behavior and the measured flow signals are stored by data acquisition devices. The sampling rate is 4000 Hz. We use the high-speed camera to observe and define oil-water flow patterns. We infer multiscale limited penetrable horizontal visibility graphs from our experimental measurements and the limited penetrable distance is 1. Then we employ the average clustering coefficient to analyze the derived complex networks corresponding to three typical vertical oil-in-water flow patterns.

**Figure 3:**
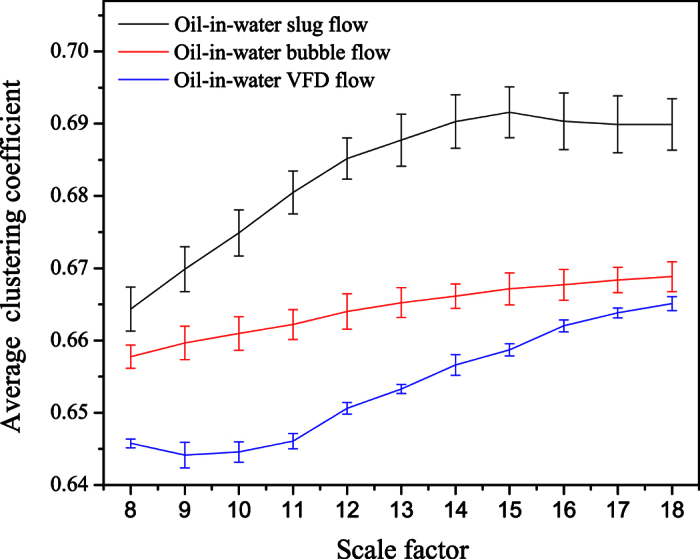


The results are shown in Fig. 3, in which each error bar is calculated from different flow conditions for the same flow pattern at the same scale factor. We can see that, the multiscale distributions of the average clustering coefficient for different flow patterns exhibit distinct features, which allows identifying three different oil-water flow patterns. For the oil-in-water slug flow, small numbers of oil droplets simultaneously follow the cap shaped oil slugs. Its flow behavior exhibits the feature of intermittent oscillation and its flow structure presents non-homogenous distribution. The flow of oil slug through the sensor will lead to a large fluctuation in the measured conductance signals. Consequently, the average clustering coefficients of oil-in-water slug flow exhibit large values at different scales, and the deviation of the average clustering coefficients calculated from different oil-in-water slug flow conditions at the same scale factor is also the largest among three flow patterns. The turbulent energy enhances with the increase of mixture flow rate, the oil slug are broken into small oil droplets consequently. That is, oil-in-water bubble flow occurs, where oil phase exists in the form of discrete droplets flowing in a water continuum. In this flow pattern, the intermittent oscillation of oil slugs gradually disappears and the non-homogenous distribution of oil phase becomes weak. The fluctuation strength of the measured conductance signals is weakened. Correspondingly, the average clustering coefficient decreases as the flow pattern changes from oil-in-water slug flow to oil-in-water bubble flow and meanwhile the deviation value also decreases. When the mixture flow rate is high, the oil droplets are dispersed into even smaller oil droplets, i.e., an onset of oil-in-water very fine dispersed bubble flow (VFD flow). The fluctuation strength of the signals from VFD flow is further weakened. The average clustering coefficient and its deviation of VFD flow are the smallest, indicating the underlying flow behavior becomes stochastic and the distribution of oil phase becomes homogenous as the flow pattern evolves from oil-in-water bubble flow to VFD flow. These interesting findings suggest that our method is capable of identifying and characterizing three typical flow patterns arising from vertical oil-water two-phase flow at different scales.

## Discussions

In summary, we have articulated a novel MLPHVG strategy (multiscale limited penetrable horizontal visibility graph) for analyzing nonlinear time series. The basic idea of MLPHVG is to define temporal scales in terms of coarse-grain process and then infer limited penetrable horizontal visibility graph from coarse-grained time series for each scale to construct MLPHVG. We choose nonlinear time series from two typical complex systems, i.e., EEG signals and two-phase flow signals, to demonstrate the effectiveness of our method. Combining MLPHVG and support vector machine, we detect epileptic seizure from two sets of EEG signals recorded from numbers of healthy subjects and epilepsy patients. The results suggest that our method allows a high-accurate classification of EEG signals recorded from healthy subjects and epilepsy patients during epileptic seizures. In addition, we use our method to derive multiscale complex network from oil-water two-phase flow signals and then employ multiscale network statistical measures to characterize the constructed networks. Our results indicate that the average clustering coefficient at different scales allows faithfully revealing the change of flow behavior underlying different flow patterns. Bridging multiscale analysis and limited penetrable horizontal visibility graph provides a novel methodology for characterizing a dynamical process underlying a given nonlinear time series of time dependent complex system which widely exists in science and engineering.

## Methods

**Figure 4:**
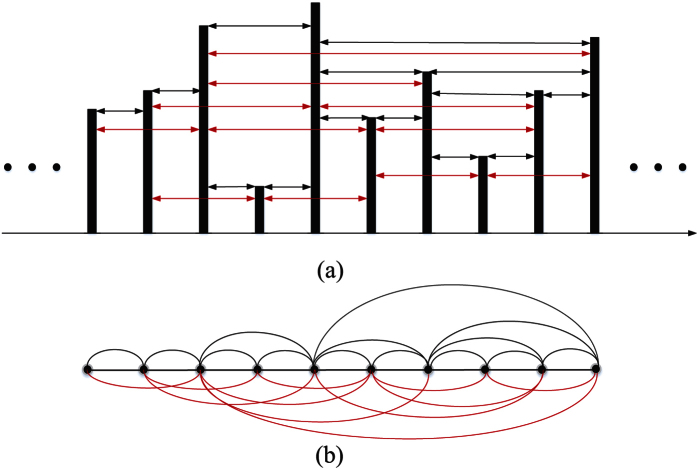
Example of (**a**) a time series (10 data values) and (**b**) its corresponding LPHVG with the limited penetrable distance *L* being 1, where every node corresponds to time series data in the same order. The horizontal visibility lines between data points define the links connecting nodes in the graph.

The multiscale limited penetrable horizontal visibility graph (MLPHVG) method can be implemented by the following steps: For a time series of length *N*, {*x* (*i*), *i* = 1, 2, …, *N*}, we first define temporal scales in terms of coarse grain process^6^ and get a coarse-grained time series {*y*^*s*^ (*j*), *j* = 1, 2, …, *N*/*s*} in the following form




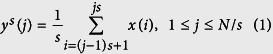




where *s* represents scale factor. Next we infer limited penetrable horizontal visibility graph from the coarse-grained time series {*y*^*s*^ (*j*), *j* = 1, 2, …, *N*/*s*}. We in Fig. 4 show a schematic diagram for demonstrating how to infer limited penetrable horizontal visibility graph from a time series. For a continuous time series of length 10, we display them in the form of vertical bars in Fig. 4(a) and regard each data point (vertical bar) as a node of a complex network. For the horizontal visibility graph (HVG)^18^, two nodes *y*^*s*^ (*i*) and *y*^*s*^ (*j*) are connected if one can draw a horizontal line joining *y*^*s*^ (*i*) and *y*^*s*^ (*j*) that does not intersect any intermediate data height. That is, a connection between two nodes *y*^*s*^ (*i*) and *y*^*s*^ (*j*) exists (black lines in Fig. 4(b)) if the following criterion is fulfilled:




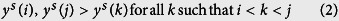




Our limited penetrable horizontal visibility graph is a development of the HVG. In particular, if we set the limited penetrable distance to *L*, a connection between two nodes exists if the number of in-between nodes that block the horizontal line is no more than *L*. As shown in Fig. 4(a,b), the red lines are the new established connections when we infer the LPHVG on the basis of HVG with the limited penetrable distance being 1. Finally, based on the above procedure, we can obtain multiscale limited penetrable horizontal visibility graph (MLPHVG) by deriving the LPHVG from the coarse-grained time series at different scales.

Representing a time series through a multiscale limited penetrable horizontal visibility graph, we can then explore the dynamic behaviors from multiscale analysis and network analysis, which is quantified via network statistical measures. In particular, we employ the average clustering coefficient (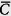)^47^, and our recently proposed clustering coefficient entropy (*E*_*C*_)^24^ to characterize the topological structure of inferred networks. These network statistical measures can be calculated as follows




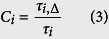







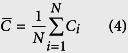







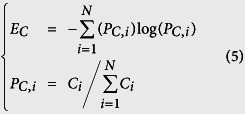




where *τ*_*i*,Δ_ denotes the number of closed triplets centered on node *i*, *τ*_*i*_ is the number of triplets centered on node *i*, *N* is the node number of the derived MLPHVG.

## Additional Information

**How to cite this article**: Gao, Z.-K. *et al.* Multiscale limited penetrable horizontal visibility graph for analyzing nonlinear time series. *Sci. Rep.* **6**, 35622; doi: 10.1038/srep35622 (2016).

## References

[b1] Daw C. S. *et al.* Self-Organization and Chaos in a Fluidized Bed. Phys. Rev. Lett. 75, 2308–2311 (1995). 10059271 10.1103/PhysRevLett.75.2308

[b2] Peng C. K. *et al.* Mosaic organization of DNA nucleotides. Phys. Rev. E 49, 1685–1689 (1994). 10.1103/physreve.49.1685 9961383

[b3] Podobnik B. & Stanley H. E. Detrended cross-correlation analysis: A new method for analyzing two nonstationary time series. Phys. Rev. Lett. 100, 084102 (2008). 18352624 10.1103/PhysRevLett.100.084102

[b4] Marwan N., Romano M. C., Thiel M. & Kurths J. Recurrence Plots for the Analysis of Complex Systems. Phys. Rep. 438, 237–329 (2007).

[b5] Lempel A. & Ziv J. On the complexity of finite sequences. IEEE Trans. Inf. Theory 22, 75–81 (1976).

[b6] Costa M., Goldberger A. L. & Peng C. K. Multiscale entropy analysis of complex physiologic time series. Phys. Rev. Lett. 89, 068102 (2002). 12190613 10.1103/PhysRevLett.89.068102

[b7] Gao Z. K., Yang Y. X., Zhai L. S., Ding M. S. & Jin N. D. Characterizing slug to churn flow transition by using multivariate pseudo Wigner distribution and multivariate multiscale entropy. Chem. Eng. J. 291, 74–81 (2016).

[b8] Liu N. R., An H. Z., Gao X. Y., Li H. J. & Hao X. Q. Breaking news dissemination in the media via propagation behavior based on complex network theory. Physica A 453, 44–54 (2016).

[b9] Kim B., Do Y. & Lai Y. C. Emergence and scaling of synchronization in moving-agent networks with restrictive interactions. Phys. Rev. E 88, 042818 (2013). 10.1103/PhysRevE.88.042818 24229236

[b10] Su R. Q., Lai Y. C., Wang X. & Do Y. Uncovering hidden nodes in complex networks in the presence of noise. Sci. Rep. 4, 3944 (2014). 24487720 10.1038/srep03944 PMC3909906

[b11] Huang Z. G., Dong J. Q., Huang L. & Lai Y. C. Universal flux-fluctuation law in small systems. Sci. Rep. 4, 6787 (2014). 25345973 10.1038/srep06787 PMC4209461

[b12] Zou W. *et al.* Restoration of rhythmicity in diffusively coupled dynamical networks. Nat. Commun. 6, 7709 (2015). 26173555 10.1038/ncomms8709 PMC4518287

[b13] Wang Z., Kokubo S., Jusup M. & Tanimoto J. Universal scaling for the dilemma strength in evolutionary games. Phys. Life Rev. 14, 1–30 (2015). 25979121 10.1016/j.plrev.2015.04.033

[b14] Gao Z. K. *et al.* Multi-frequency complex network from time series for uncovering oil-water flow structure. Sci. Rep. 5, 8222 (2015). 25649900 10.1038/srep08222 PMC4316157

[b15] Zhang J. & Small M. Complex network from pseudoperiodic time series: topology versus dynamics. Phys. Rev. Lett. 96, 238701 (2006). 16803415 10.1103/PhysRevLett.96.238701

[b16] Xu X. K., Zhang J. & Small M. Superfamily phenomena and motifs of networks induced from time series. P. Natl. Acad. Sci. USA 105, 19601–19605 (2008). 10.1073/pnas.0806082105 PMC2604928 19064916

[b17] Lacasa L., Luque B., Ballesteros F., Luque J. & Nuno J. C. From time series to complex networks: The visibility graph. P. Natl. Acad. Sci. USA 105, 4972–4975 (2008). 10.1073/pnas.0709247105 PMC2278201 18362361

[b18] Luque B., Lacasa L., Ballesteros F. & Luque J. Horizontal visibility graphs: exact results for random time series. Phys. Rev. E 80, 046103 (2009). 10.1103/PhysRevE.80.046103 19905386

[b19] Marwan N., Donges J. F., Zou Y., Donner R. V. & Kurths J. Complex network approach for recurrence analysis of time series. Phys. Lett. A 373, 4246–4254 (2009).

[b20] Gao Z. K. & Jin N. D. A directed weighted complex network for characterizing chaotic dynamics from time series. Nonlinear Anal.-Real 13, 947–952 (2012).

[b21] Donges J. F., Heitzig J., Donner R. V. & Kurths J. Analytical framework for recurrence network analysis of time series. Phys. Rev. E 85, 046105 (2012). 10.1103/PhysRevE.85.046105 22680536

[b22] Gao Z. K., Fang P. C., Ding M. S. & Jin N. D. Multivariate weighted complex network analysis for characterizing nonlinear dynamic behavior in two-phase flow. Exp. Therm. Fluid Sci. 60, 157–164 (2015).

[b23] Gao Z. K. *et al.* Recurrence networks from multivariate signals for uncovering dynamic transitions of horizontal oil-water stratified flows. Europhys. Lett. 103, 50004 (2013).

[b24] Gao Z. K. *et al.* Multiscale complex network for analyzing experimental multivariate time series. Europhys. Lett. 109, 30005 (2015).

[b25] Huang S. P., An H. Z., Gao X. Y. & Jiang M. H. The multiscale fluctuations of the correlation between oil price and wind energy stock. Sustainability 8, 534 (2016)

[b26] Lacasa L. & Toral R. Description of stochastic and chaotic series using visibility graphs. Phys. Rev. E 82, 036120 (2010). 10.1103/PhysRevE.82.036120 21230152

[b27] Luque B., Lacasa L., Ballesteros F. J. & Robledo A. Feigenbaum graphs: a complex network perspective of chaos. PLoS One 6, e22411 (2011). 21915254 10.1371/journal.pone.0022411 PMC3168432

[b28] Liu C., Zhou W. X. & Yuan W. K. Statistical properties of visibility graph of energy dissipation rates in three-dimensional fully developed turbulence. Physica A 389, 2675–2681 (2010).

[b29] Gao Z. K., Du M., Hu L. D., Zhou T. T. & Jin N. D. Visibility graphs from experimental three phase flow for characterizing dynamic flow behavior. Int. J. Mod. Phys. C 23, 1250069 (2012).

[b30] Gao X. Y. *et al.* Characteristics of the transmission of autoregressive sub-patterns in financial time series. Sci. Rep. 4, 6290 (2014). 25189200 10.1038/srep06290 PMC4155334

[b31] Ravetti M. G., Carpi L. C., Goncalves B. A., Frery A. C. & Rosso O. A. Distinguishing Noise from Chaos: Objective versus Subjective Criteria Using Horizontal Visibility Graph. PLoS One 9, e108004 (2014). 25247303 10.1371/journal.pone.0108004 PMC4172653

[b32] Zhuang E., Small M. & Feng G. Time series analysis of the developed financial markets’ integration using visibility graphs. Physica A 410, 483–495 (2014).

[b33] Zou Y., Donner R. V., Marwan N., Small M. & Kurths J. Long-term changes in the north-south asymmetry of solar activity: a nonlinear dynamics characterization using visibility graphs. Nonlinear Proc. Geoph. 21, 1113–1126 (2014).

[b34] Tang J. J., Liu F., Zhang W. B., Zhang S. & Wang Y. H. Exploring dynamic property of traffic flow time series in multi-states based on complex networks: Phase space reconstruction versus visibility graph. Physica A 450, 635–648 (2016).

[b35] Stephen M., Gu C. G. & Yang H. J. Visibility Graph Based Time Series Analysis. PLoS One 10, e0143015 (2015). 26571115 10.1371/journal.pone.0143015 PMC4646626

[b36] Lucas L., Vincenzo N. & Vito L. Network structure of multivariate time series. Sci. Rep. 5, 15508 (2015). 26487040 10.1038/srep15508 PMC4614448

[b37] Zhang B., Wang J. & Fang W. Volatility behavior of visibility graph EMD financial time series from Ising interacting system. Physica A 432, 301–314 (2015).

[b38] Bhaduri S. & Ghosh D. Electroencephalographic data analysis with visibility graph technique for quantitative assessment of brain dysfunction. Clin. EEG Neurosci. 46, 218–223 (2015). 24781371 10.1177/1550059414526186

[b39] Zhu G., Li Y. & Wen P. Analysis of alcoholic EEG signals based on horizontal visibility graph entropy. Brain Informatics 1, 19–25 (2014). 27747525 10.1007/s40708-014-0003-x PMC4883153

[b40] Ahmadlou M., Adeli H. & Adeli A. New diagnostic EEG markers of the Alzheimer’s disease using visibility graph. J. Neural Transm. 117, 1099–1109 (2010). 20714909 10.1007/s00702-010-0450-3

[b41] Zhou T. T., Jin N. D. Gao Z. K. & Luo Y. B. Limited penetrable visibility graph for establishing complex network from time series. Acta Phys. Sin. 61, 030506 (2012).

[b42] Gao Z. K., Hu L. D., Zhou T. T. & Jin N. D. Limited penetrable visibility graph from two-phase flow for investigating flow pattern dynamics. Acta Phys. Sin. 62, 110507 (2013).

[b43] Wang R. X., Gao J. M., Gao Z. Y., Gao X. & Jiang H. Q. Complex network theory-based condition recognition of electromechanical system in process industry. Sci. China Technol. Sc. 59, 604–617 (2016).

[b44] Pei X. *et al.* WLPVG approach to the analysis of EEG-based functional brain network under manual acupuncture, Cogn. Neurodynamics 8, 417–428 (2014). 10.1007/s11571-014-9297-x PMC4155065 25206935

[b45] Wang J. *et al.* Functional brain networks in Alzheimer’s disease: EEG analysis based on limited penetrable visibility graph and phase space method. Physica A 460, 174–187 (2016).

[b46] Andrzejak R. G. *et al.* Indications of nonlinear deterministic and finite-dimensional structures in time series of brain electrical activity: Dependence on recording region and brain state. Phys. Rev. E 64, 061907 (2001). 10.1103/PhysRevE.64.061907 11736210

[b47] Newman M. E. J. The structure and function of complex networks. SIAM Rev. 45, 167–256 (2003).

[b48] An S. J., Liu W. Q. & Venkatesh S. Fast cross-validation algorithms for least squares support vector machine and kernel ridge regression. Pattern Recogn. 40, 2154–2162 (2007).

[b49] Nigam V. P. & Graupe D. A neural network based detection of epilepsy. Neurol. Res. 26, 55–60 (2004). 14977058 10.1179/016164104773026534

[b50] Kaya Y., Uyar M., Tekin R. & Yildirim S. 1D-local binary pattern based feature extraction for classification of epileptic EEG signals. Appl. Math. Comput. 243, 209–219 (2014).

[b51] Kannathal N., Choo M. L., Acharya U. R. & Sadasivan P. K. Entropies for detection of epilepsy in EEG. J. Med. Syst. 80, 187–194 (2005). 10.1016/j.cmpb.2005.06.012 16219385

[b52] Subasi A. EEG signal classification using wavelet feature extraction and a mixture of expert model. Expert Syst. Appl. 32, 1084–1093 (2007).

[b53] Polat K. & Gunes S. Classification of epileptiform EEG using a hybrid system based on decision tree classifier and fast Fourier transform. Appl. Math. Comput. 187, 1017–1026 (2007).

[b54] Nicolaou N. & Georgiou J. Detection of epileptic electroencephalogram based on Permutation Entropy and Support Vector Machines. Expert Syst. Appl. 39, 202–209 (2012).

[b55] Zhu G. H., Li Y. & Wen P. Epileptic seizure detection in EEGs signals using a fast weighted horizontal visibility algorithm. Comput. Meth. Prog. Bio. 115, 64–75 (2014). 10.1016/j.cmpb.2014.04.001 24768081

[b56] Zamir Z. R. Detection of epileptic seizure in EEG signals using linear least squares preprocessing. Comput. Methods Programs Biomed. 133, 95–109 (2016). 27393803 10.1016/j.cmpb.2016.05.002

[b57] Acharya U. R., Fujita H., Sudarshan V. K., Bhat S. & Koh J. E. W. Application of entropies for automated diagnosis of epilepsy using EEG signals: A review. Knowl-Based Syst. 88, 85–96 (2015).

[b58] Gao Z. K., Yang Y. X., Zhai L. S., Jin N. D. & Chen G. R. A four-sector conductance method for measuring and characterizing low-velocity oil-water two-phase flows. IEEE Transactions on Instrumentation and Measurement 65, 1690–1697 (2016).

